# Expression and Purification of Glycosyltransferase DnmS from *Streptomyces peucetius* ATCC 27952 and Study on Catalytic Characterization of Its Reverse Glycosyltransferase Reaction

**DOI:** 10.3390/microorganisms11030762

**Published:** 2023-03-16

**Authors:** Liyan Yang, Huimin Zhou, Guiguang Chen, Hongliang Li, Dengfeng Yang, Lixia Pan

**Affiliations:** 1 State Key Laboratory of Non-Food Biomass and Enzyme Technology, Guangxi Key Laboratory of Marine Natural Products and Combinatorial Biosynthesis Chemistry, Guangxi Academy of Sciences, Nanning 530007, China; 2 College of Life Science and Technology, Guangxi University, Nanning 530004, China; 3 Institute of Biology, Guangxi Academy of Sciences, Nanning 530007, China; 4 College of Food and Quality Engineering, Nanning University, Nanning 530200, China

**Keywords:** glycosyltransferase, fusion protein, DnmS, DnmQ, chaperone plasmids, co-expression, reverse glycosyltransfer

## Abstract

Anthracyclines are an important class of natural antitumor drugs. They have a conservative aromatic tetracycline backbone that is substituted with different deoxyglucoses. The deoxyglucoses are crucial for the biological activity of many bacterial natural products after the proper modification from glycosyltransferases (GTs). The difficulty in obtaining highly purified active GTs has prevented biochemical studies on natural product GTs. In this paper, a new *Escherichia coli* fusion plasmid pGro7′, which introduces the *Streptomyces coelicolor* chaperone genes *groEL1*, *groES* and *groEL2,* was constructed. The glycosyltransferase DnmS from *Streptomyces peucetius* ATCC 27952 was co-expressed with the plasmid pGro7′, and unprecedented high-efficiency and soluble expression of DnmS in the *E. coli* expression system was realized. Subsequently, the reverse glycosylation reaction characteristics of DnmS and DnmQ were verified. We found that DnmS and DnmQ had the highest enzyme activity when they participated in the reaction at the same time. These studies provide a strategy for the soluble expression of GTs in *Streptomyces* and confirm the reversibility of the catalytic reaction of GTs. This provides a powerful method for the production of active anthracyclines and to enhance the diversity of natural products.

## 1. Introduction

*Streptomyces peucetius* ATCC 27952 is a filamentous soil bacterium with the potential to produce anthracyclines, such as doxorubicin (DXR) [1,2,3,4], daunorubicin (DNR) [5] and other anthracycline antibiotics. Among them, DNR and DXR were isolated from the fermentation broth of *Streptomyces* in the 1960s. They have inhibitory effects on leukemia, lymphoid system tumors and a variety of solid tumors. At present, they have become the first-line antitumor drugs in clinical practice [6].

The anthraquinone moiety of the anthracyclines intercalates between DNA bases, while the carbohydrate unit at the 7-position enhances binding through interactions with the minor groove of DNA [7]. Structural biology studies have shown that the deoxy sugar group can closely bind to the DNA groove, which has an important impact on the antitumor effects of anthracycline natural products [8].

The four-ring skeleton structure of DNR is formed by the condensation reaction of type II polyketide synthase with malonyl-CoA as the starting unit and malonyl-CoA as the extension unit. Then, anthracyclines feature O-glycosides primarily at phenolic oxygens at the 7-position. After demethylation, methylation and oxidation, DNR products are formed [9,10]. DXR is the product of further hydroxylation at the C-14 position of DNR, which has a stronger antitumor effect [11,12]. The anthracyclines suffer from dose-limiting cardiotoxicity [13], which has motivated efforts to study the biosynthesis of anthracyclines and to generate new drug analogues with improved clinical properties.

The combination of DNA and chromatin damage has been shown to be the main cause of cardiotoxicity of DXR [14]. Therefore, it is very important to modify the sugar moiety to produce anthracycline drugs with less toxicity. Glycosyltransferases (GTs) are a large family of structurally conserved enzymes responsible for catalyzing the transfer of a sugar moiety from an activated donor sugar to an acceptor molecule [15]. GTs can transfer different sugar fractions to produce active anthracycline derivatives. However, for most GTs, it is difficult to express soluble and effective in vitro, and there is a lack of research on transforming anticancer drugs with GTs in vitro. Therefore, in order to obtain low toxicity and effective anticancer drugs, the soluble expression of GTs is imminent.

Most GTs from actinomycetes are usually expressed in *E. coli*; however, this process usually produces inclusion, which not only limits the expression of recombinant proteins but also limits the transformation of anticancer drugs [16]. In the process of the anthracycline DXR biosynthesis pathway, DnmS is responsible for glycosylation modification of the C-7 hydroxyl group of the ε-rhodomycinone (ε-RHO) skeleton to form a monoglycosylaglycone substituted intermediate rhodomycin D (RHOD), DnmQ assists DnmS to complete its catalysis.

The translated gene sequences for DnmQ share moderate homology with cytochrome P450 enzymes, yet lack the conserved Cys residue that coordinates the heme iron center. Researchers have attempted to express and purify DnmS and DnmQ; however, they could not solve the problem of soluble expression [17,18]. Therefore, the purpose of this experimental study is to solve the soluble expression of DnmS and its accessory protein DnmQ in *E. coli*. This lays a foundation for further study on the structure of DnmS and DnmQ and the transformation of new anthracycline drugs. In this research, we first screened a variety of protein expression hosts and vectors, and we then found that recombinant plasmids, including pET32a-dnmS and pET32a-dnmQ, were slightly solubilizing in *E. coli* BL21 (DE3) heterologous expression hosts.

Then, we investigated the *E. coli* chaperone plasmid pGro7 and found that, although the co-expression of the target protein was somewhat improved in the above *E. coli* chaperone plasmid, the expression level of the target protein was not sufficient for in vitro studies [19]. In order to solve this problem, we had to improve the efficient and soluble expression of the target protein. Later, we learned that DnmS and DnmQ are homologous to EryCIII and EryCII, which are responsible for the biosynthesis of erythromycin D. They are involved in the glycosylation of erythromycin D. The expression of EryCIII in the *E. coli* BL21 (DE3) codon plus RP cells yielded insoluble protein.

Co-expression with the *E. coli* chaperonins GroEL and GroES yielded some soluble protein; however, purification was compromised by contaminating GroEL and GroES. In contrast, co-expression with the *Streptomyces coelicolor* chaperonins GroEL1, GroES and GroEL2 produced soluble protein that could be purified to homogeneity [20,21,22,23]. This gave us an idea to solve the problem of high-efficiency protein expression. We deleted the self-chaperone genes *groEL* and *groES* from the existing *E. coli* molecular chaperone plasmid pGro7, which was obtained from a commercial source and then introduced the *Streptomyces coelicolor* chaperonin genes *groEL1*, *groES* and *groEL2*—that is, the modified pGro7′. The results showed that the chaperone plasmid pGro7′ helped the expression of the target protein DnmS more efficiently than in plasmid pGro7. This discovery has great commercial potential and provides the possibility for the crystallization and structural study of DnmS.

In this experiment, the highly expressed soluble protein DnmS was obtained by using the modified chaperone plasmid pGro7′ for the first time. As the DnmQ assisted DnmS to complete its catalysis, we also expressed DnmQ successfully using expression vector pET32a in this study.

We learned that most GTs can also use nucleoside diphosphate (NDP) to hydrolyze glycosylation in natural product molecules—that is, a reversible glycosylation reaction participated in by GTs [24]. For example, four GTs from two different natural product biosynthesis pathways, calimycin and vancomycin, can easily catalyze the reversible reaction, making it easy to exchange sugars and aglycones. This indicates that the reversibility of GT-catalyzed reactions may be general and useful for generating exotic nucleotide sugars, thus, establishing in vitro GT activity in complex systems and enhancing natural product diversity [24].

In this experiment, soluble DnmS and DnmQ were obtained, and commercial DNR was used as a substrate. The reverse glycosyltransfer reactions catalyzed by DnmS, DnmQ and DnmS/DnmQ mixed protein were tested in vitro. Among the different enzyme reactions, DnmS/DnmQ mixed protein had the highest enzyme activity. These works verified the reverse catalytic mechanism of DnmS for the first time.

These works not only expand the understanding of the post-modification process of DXR biosynthesis but also provide guidance for the generation of more structurally diverse anthracycline derivatives.

## 2. Materials and Methods

### 2.1. Strains and Materials

*E. coli* strains were grown at 37 °C in Luria–Bertani (LB) media in both liquid and agar plates supplemented with the appropriate amount of antibiotic. *S. peucetius* was grown in solid MS medium (mannitol 20 g/L, soya flour 20 g/L and agar 20 g/L) with appropriate antibiotics for recombinant strains at 28 °C for 5 days. *E. coli* DH5α was used for recombinant plasmid construction. *E. coli* BL21 (DE3) was used as the protein expression host. The plasmids pET22b and pET32a (Table 1) were used as expression vectors. Antibiotics were added at the following concentrations for *E. coli*: kanamycin (Kan) 50 μg/mL; ampicillin (Amp) 100 μg/mL; and chloramphenicol (Cm) 34 μg/mL. The following supplement was added when required: isopropyl-β-D-thiogalactopyranoside (IPTG) 0.1 mM.

### 2.2. Molecular Cloning and Construction of Recombinant Plasmids

The *dnmS* and *dnmQ* genes were derived from *Streptomyces peucetius* ATCC 27952 (GenBank: L47164.1). *dnmS* and *dnmQ* genes were amplified with the primer sets 22b-dnmS-F/R, 32a-dnmS-F/R, 22b-dnmQ-F/R and 32a-dnmQ-F/R (Table 2). The total DNA of the *Streptomyces* strain was used as the PCR template. The PCR products were purified with a PCR clean-up kit according to manufacturer’s description. The purified *dnmS* fragment was ligated into pET32a, which was digested with BamH I and Hind III using the ClonExpress II One Step Cloning Kit (Vazyme, China) to generate the recombinant plasmid named 32aS (Table 1). The plasmids 22bS, 22bQ and 32aQ (Table 1) were constructed using the same strategy. The plasmids 22bS, 32aS, 22bQ and 32aQ were transformed into expression hosts BL21 (DE3) and BL21 Codon plus (DE3) RIL generating the strains 22bS/DE3, 22bS/RIL, 32aS/DE3, 32aS/RIL, 22bQ/DE3, 22bQ/RIL, 32aQ/DE3 and 32aQ/RIL.

**Figure 1:**
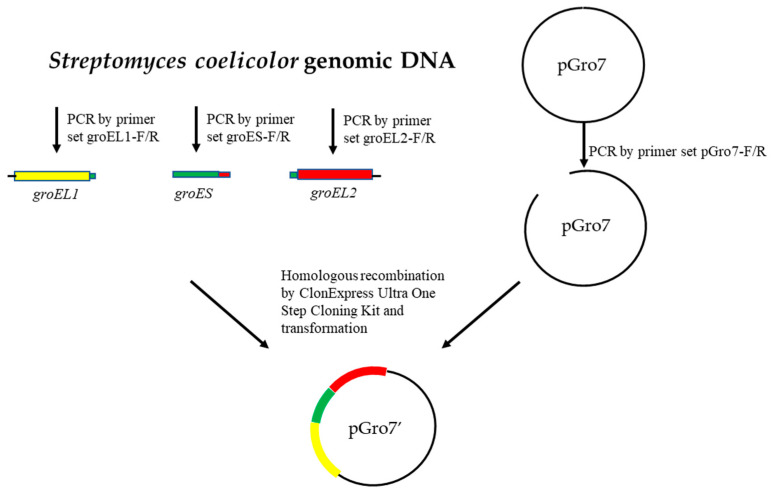
Construction process for the pGro7′ plasmid.

The *groEL1*, *groES* and *groEL2* genes were derived from *Streptomyces coelicolor* (GenBank: AL645882.2). As shown in Figure 1, the construction of plasmid pGro7′ was as follows: the DNA fragment of *groEL1* was obtained through PCR using the primer set groEL1-F/R, which has a homologous arm of pGro7 at the 5′ end and a homologous arm of *groES* at 3′ end. The DNA fragment of groES was obtained through PCR using the primer set groES-F/R, which has a homologous arm of *groEL2* at the 3′ end. The DNA fragment of *groEL2* was obtained through PCR using the primer set groEL2-F/R, which has a homologous arm of *groES* at the 5′ end and a homologous arm of pGro7at the 3′ end, and a pGro7 fragment lacking the groEL and groES genes was obtained through PCR using the primer set pGro7-F/R (Table 2). Then, these fragments were connected by homologous recombination using ClonExpress Ultra One Step Cloning Kit (Vazyme, China) to generate the chaperone plasmid pGro7′ with the araB promoter (Figure 1). The plasmid 32aS and chaperone plasmid pGro7′ were co-transformed into BL21(DE3) to generate the strain 32aS/pGro7′.

### 2.3. Overexpression of dnmS and dnmQ in E. coli Strains

For the protein overexpression, 500 mL of LB supplemented with 2 mg/mL of L-arabinose as an inducer for the expression of the chaperons GroES/EL was inoculated with 5 mL of an overnight seed culture grown from a single colony. Cells were grown at 37 °C to OD_600_ of about 0.6–0.8 and then induced by adding IPTG to a final concentration of 0.1 mM. The cells were incubated for 24 h at 16 °C. The cell pellets were harvested by centrifugation at 7000× *g* for 15 min and resuspended with buffer containing 50 mM Tris, 300 mM NaCl and 10% glycerol at pH 7.5. Finally, the cell pellets were lysed by ultra-sonication. The soluble protein was separated from the cell debris by centrifugation at 12,000 rpm for 30 min at 4 °C. Other strains were expressed using the same strategy.

### 2.4. Purification and Isolation of DnmS and DnmQ

After centrifugation, the supernatant was loaded onto a column containing HisPur™ Ni-NTA Resin (GE Healthcare, Chicago, IL, USA) for His-tag affinity purification. The column was washed five times with wash buffer (50 mM Tris, 300 mM NaCl, 10% glycerol and 50 mM imidazole at pH 7.5) to remove contaminating proteins. The target protein was eluted with elution buffer (50 mM Tris, 300 mM NaCl, 10% glycerol and 500 mM imidazole at pH 7.5). The finally obtained protein was analyzed using 12% sodium dodecyl sulfate polyacrylamide gel electrophoresis (SDS-PAGE).

### 2.5. Enzyme Assay of DnmS and DnmQ

After obtaining DnmS and DnmQ soluble proteins, we began to study the catalytic function of DnmS and the auxiliary protein DnmQ in vitro. We first used DNR as the reaction substrate to measure the enzyme activity of the reverse glycosyltransfer reactions of DnmS alone, DnmQ alone and DnmS/DnmQ mixed protein. The enzyme used in the reaction system was the supernatant of the bacterial liquid after cell destruction.

The total volume of the performed test was 100 µL at pH 7.5 of 10 mM Tris-HCl and 1 mM MgCl_2_ buffer, and 2 mM thymidine diphosphate (TDP), 50 µM DNR and 100 µM total protein were added to the system at the same time. The reaction was conducted at 30 °C for 12 h. An equal volume of methanol was added to terminate the enzyme reaction with a 12,000 rpm centrifuge for 20 min to remove denatured protein. The reaction product was detected by HPLC (KromaSil C18, 5 µm, 250 × 4.6 mm, 0.1% trifluoroacetic acid (TFA) in H_2_O with a 10–100% CH_3_CN gradient over 20 min at 1 mL/min; A280), and the products were confirmed by liquid chromatography-mass spectrometry (LC-MS).

## 3. Results

### 3.1. The Expression of DnmQ in pET22b and pET32a

**Figure 2:**
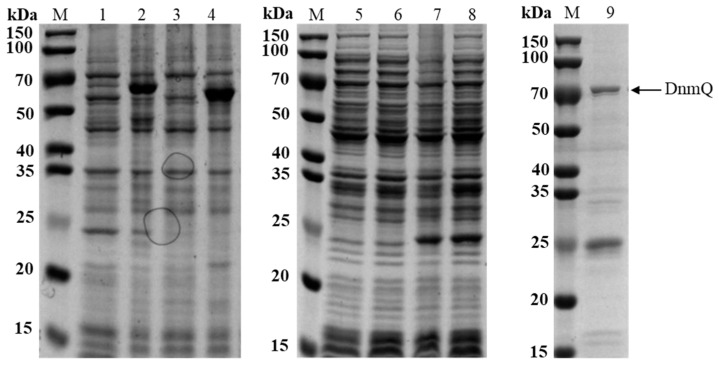
The expression of DnmQ in different expression vectors and hosts. Lane 1, 22bQ/DE3 induced supernatant; Lane 2, 22bQ/DE3 induced total protein; Lane 3, 22bQ/RIL induced supernatant; Lane 4, 22bQ/RIL induced total protein; Lane 5, 32aQ/DE3 induced supernatant; Lane 6, 32aQ/DE3 induced total protein; Lane 7, 32aQ/RIL induced supernatant; Lane 8, 32aQ/RIL induced total protein; and Lane 9, DnmQ purified from 32aQ/DE3; M, protein marker (Genestar, 15, 20, 25, 35, 40, 50, 70, 100 and 150 kDa.).

*dnmQ* gene was cloned into pET22b, which contains C-His6-tag, and pET32a, which contains N-His6-tag, and transformed into expression hosts BL21 (DE3) and BL21 Codon plus (DE3) RIL. The C-His6-tagged fusion protein DnmQ was almost in inclusion bodies, while the N-His6-tagged fusion protein DnmQ was expressed well (Figure 2).

### 3.2. The Expression of DnmS in Different Expression Vectors and Hosts

In order to express DnmS protein, the *dnmS* gene was first cloned into pET22b, which contains C-His_6_-tag, and was transformed into expression hosts BL21 (DE3) and BL21 Codon plus (DE3) RIL. The C-His6-tagged fusion protein DnmS was almost in inclusion bodies (Figure S1). In order to obtain the soluble expression of *dnmS*, we attempted to clone the *dnmS* gene into pET32a, which contains N-His6-tag and Trx tag, and transformed it into expression hosts BL21 (DE3) and BL21 Codon plus (DE3) RIL. However, DnmS was still in the inclusion body (Figure S1).

### 3.3. Enhanced Soluble Expression of DnmS in E. coli BL21 (DE3) by Chaperone Plasmid

**Figure 3:**
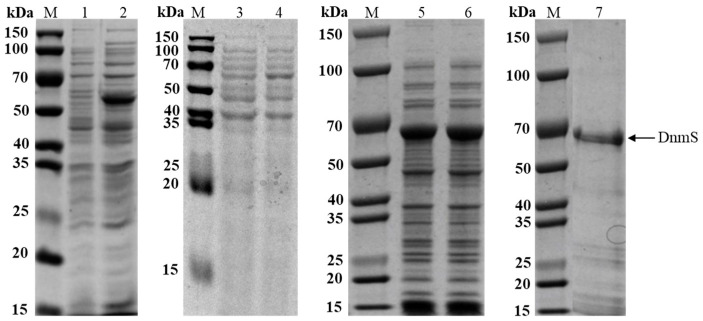
Expression of DnmS with different fusion partners in *E. coli*. Lane 1, 32aS/DE3 induced supernatant; Lane 2, 32aS/DE3 induced total protein; Lane 3, 32aS/pGro7 induced supernatant; Lane 4, 32aS/pGro7 induced total protein; Lane 5, 32aS/pGro7′ induced supernatant; Lane 6, 32aS/pGro7′ induced total protein; and Lane 7, DnmS purified from 32aS/pGro7′; M, protein marker (Genestar, 15, 20, 25, 35, 40, 50, 70, 100 and 150 kDa.).

The plasmid 32aS and *E. coli* chaperone plasmid pGro7 were co-expressed in expression host BL21 (DE3) to obtain soluble DnmS protein. The soluble expression of the recombinant protein was somewhat improved with the help of *E. coli* chaperone plasmid pGro7, and the soluble expression rate was 28% (as determined by ImageJ); however, this expression level of DnmS was not sufficient for studies in vitro (Figure 3).

In order to solve this problem, we needed to improve the efficient and soluble expression of the target protein. Finally, we used the pET32a plasmid and *E. coli* BL21 (DE3) expression host and attempted to use *Streptomyces* chaperone plasmid pGro7′. Compared with *E. coli* chaperone plasmid pGro7, pGro7′ can more effectively help the soluble expression of DnmS (Figure 3), and the soluble expression rate reached 54% (as determined by ImageJ). It may be that the three chaperone genes *groEL1*, *groES* and *groEL2* in pGro7′ and *dnmS* are derived from *Streptomyces*. The recombinant enzyme was analyzed by SDS-PAGE. A significant band was observed at approximately 64 kDa.

### 3.4. Reverse Catalytic Reaction of DnmS and DnmQ In Vitro

It was reported that glycosyltransferases have reversible reactions [24]. We speculated on the reverse glycosyltransfer reaction of DnmS as follows:

Pathway A: (a) DNR receives two electrons to obtain divalent anion intermediate 2. (b) Then, under the action of DnmS and TDP, the next two protons coordinated with 2 will produce intermediate 3, which is transferred to intermediate 4 through a series of double bonds. (c) Intermediate 4 is attacked by water molecules to obtain intermediate 5, and the carbonyl group of the ketone part of 5 is attacked by hydrogen free radicals to form intermediate 6. (d) Finally, intermediate 6 receives an electron and a proton to obtain product 8.

**Figure 4:**
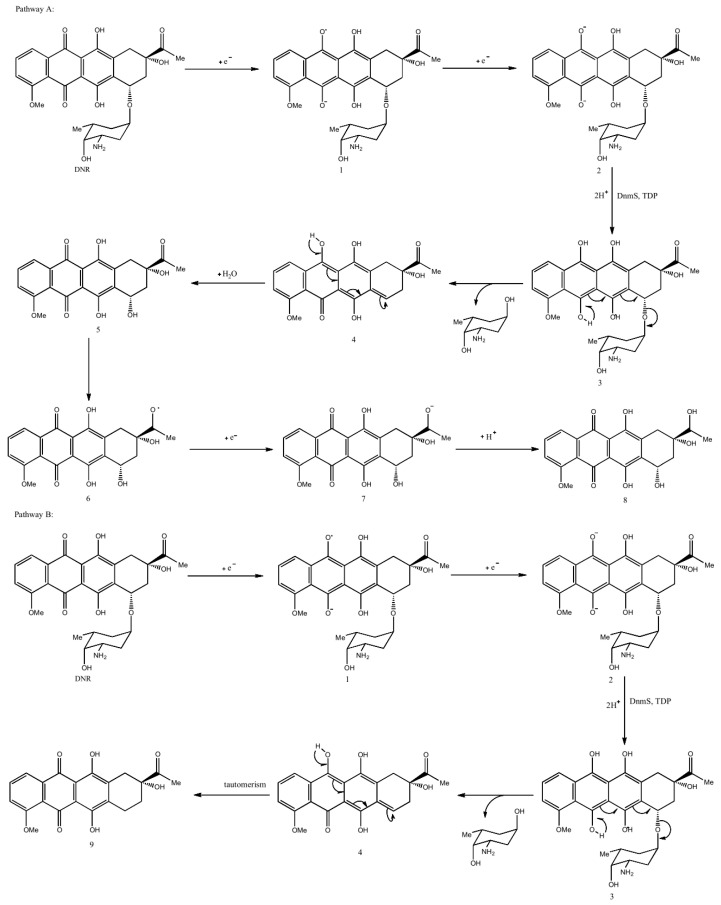
Pathway A and B show DnmS catalyzed reverse glycosyltransfer reactions and possible electron and free radical transfer during the reaction. Pathway A: DNR receives two electrons to obtain divalent anion intermediate 2. Then, under the action of DnmS and TDP, the next two protons coordinated with 2 produce intermediate 3, which is transferred to intermediate 4 through a series of double bonds. Intermediate 4 is attacked by water molecules to obtain intermediate 5, and the carbonyl group of the ketone part of 5 is attacked by hydrogen free radicals to form intermediate 6. Finally, intermediate 6 receives an electron and a proton to obtain product 8. Pathway B: DNR receives two electrons and gives the dianion intermediate 2. Then, under the action of DnmS and TDP, the subsequent two protons coordinating with 2 produce intermediate 3, which gives intermediate 4 via a series of double bond migrations. Tautomerism of 4 generates product 9 with a 1,7-H migration.

Pathway B: (a) DNR receives two electrons and gives the dianion intermediate 2. (b) Then, under the action of DnmS and TDP, the subsequent two protons coordinating with 2 produce intermediate 3, which gives intermediate 4 via a series of double bond migrations. (c) Tautomerism of 4 generates product 9 with a 1,7-H migration. (Figure 4).

**Figure 5:**
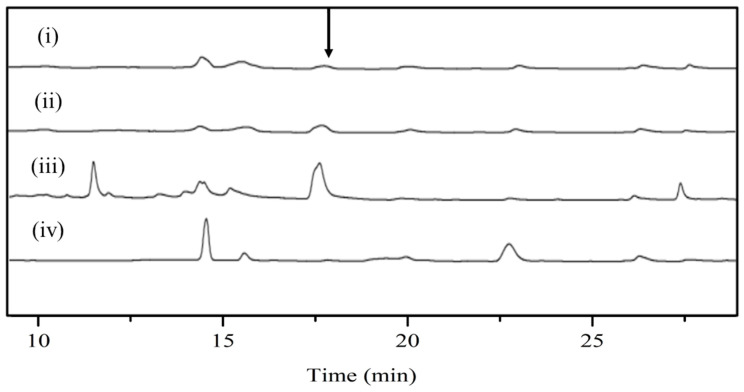
HPLC analysis of DnmSQ-catalyzed reverse glycosyltransfer reaction. All enzyme assays were performed in a total volume of 100 µL in Tris-HCl buffer (10 mM, pH 7.5) containing 1 mM of MgCl_2_ with incubation at 30 °C for 12 h. The enzyme used in the reverse glycosyltransfer reactions here refers to the cell lysate supernatant. (i) 100 µM DnmQ, 50 µM DNR and 2 mM TDP; (ii) 100 µM DnmS, 50 µM DNR and 2 mM TDP; (iii) co-incubation of 100 µM DnmS and 30 µM DnmQ, 50 µM DNR and 2 mM TDP; and (iv) control with 50 µM DNR and 2 mM TDP in the absence of enzyme resulted in no reaction. The black arrow indicates the new peak.

**Figure 6:**
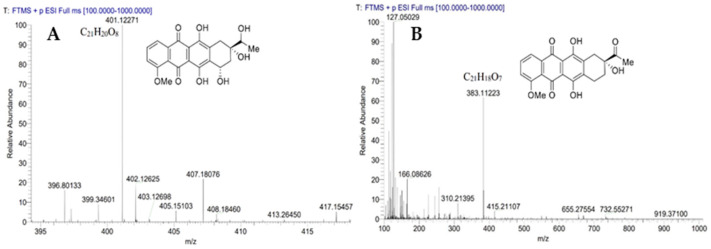
LC-MS analysis of DnmSQ-catalyzed reactions. (**A**) LC-MS compound 8 and (**B**) LC-MS compound 9.

In order to determine the reverse glycosyltransfer activity of DnmS, three enzyme reactions, including DnmS protein alone, DnmQ protein alone and DnmS/DnmQ mixed protein, were performed in this study. The DNR was used as the substrate, and the reaction product was analyzed using HPLC and LC-MS. The results of HPLC showed that there was a product in the reaction of DnmS protein alone and DnmS/DnmQ mixed protein at 17.6 min, while there was no corresponding peak in the reaction of DnmQ protein alone and in the control (Figure 5). The products in the reaction of DnmS/DnmQ mixed protein were significantly more than those found for DnmS protein alone (Figure 5). This indicates that DnmS has higher catalytic efficiency in the presence of DnmQ. The products were subsequently identified as compounds 8 and 9 by LC-MS (Figure 6).

### 3.5. Sequence Analysis, 3D-Structure Prediction and Molecular-Docking Studies of DnmS

In order to further study the mechanism of DnmS, we determined the key amino acid sites of DnmS through the homologous amino acid sequence. For example, in calicheamicin glycosyltransferase CalG2, the imidazole moiety of the equivalent histidine residue (H322) and the glycine residues G324 and G326 are involved in pyrophosphate recognition. T327 of CalG2 participates in phosphate and sugar recognition [25].

**Figure 7:**
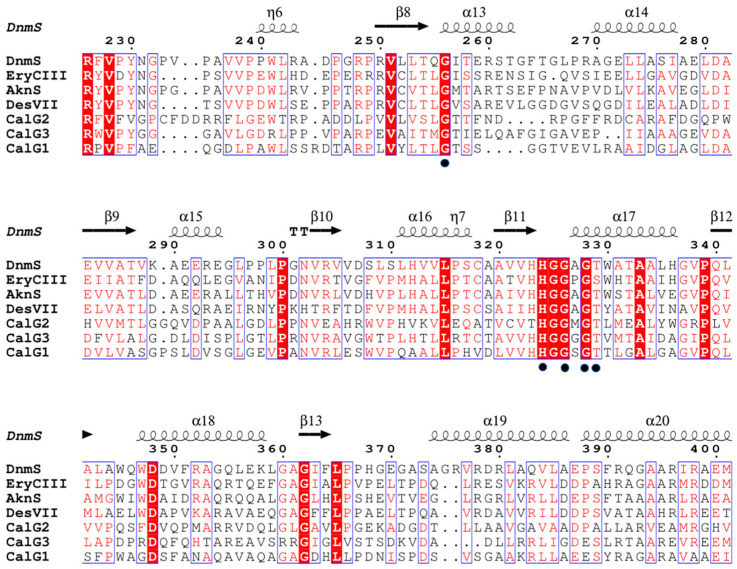
DnmS homologous sequence alignment. EryCIII (AAB84067.1 PDB entry: 2YJN) from *Saccharopolyspora erythraea* involved in the biosynthesis of erythromycin; AknS AF264025 involved in the biosynthesis of the macrolide antibiotic aclacinomycin A; DesVII (AAC68677.1) involved in the biosynthesis of the macrolide antibiotics methymycin, neomethymycin, narbomycin and pikromycin; CalG1 (PDB entry: 3OTG), CalG2 (PDB entry: 3IAA) and CalG3 (PDB entry: 3D0Q) from *Micromonospora echinospora* involved in the biosynthesis of enediynecalicheamicin. Secondary structural elements in the structure of DnmS are represented above the multiple-sequence alignment. The highly conserved residues are shaded in red, residues not fully conserved are represented by blue boxes, and comparative residues are indicated by black circles. Multiple-sequence alignment was performed using ClustalX and edited with ESPript.

Similarly, H324, G326/328 and T329 in DnmS are absolutely conserved in the GTs shown (Figure 7), and an analogous interaction is expected between the phosphate and sugar recognition of DnmS. G255 of EryCIII is conserved in the GTs, and the equivalent residue in CalG2 interacts with the pyrophosphate oxygen atoms O2A, O3A and C5M on thymidine [25]. At the same time, homologous amino acid sequence alignment shows that the amino acid at this position of DnmS is also conserved, suggesting that G256 of DnmS may have the same effect (Figure 7).

However, for most GTs, their functions are still unknown [26]. Thus, in the absence of protein crystal structures, comparative homology modelling is an excellent tool to study protein function [27,28]. The binding mode of the donor and receptor will affect the catalytic activity of GTS. In order to further explore the binding sites between DnmS and substrate, the structure of DnmS was predicted by AlphaFold2, and Autodock 4.2.6 software was used to realize the molecular docking of DnmS with two different substrates, DNR and TDP, to finally assess their stereochemical properties and side-chain environments.

**Figure 8:**
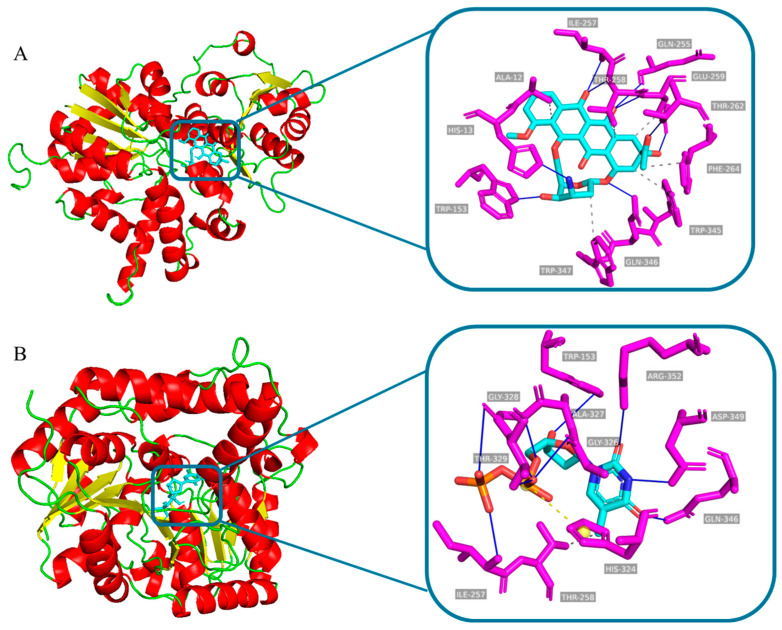
Molecular docking of DNR and TDP with our DnmS model. (**A**) DNR docking in the binding site of DnmS. (**B**) TDP docking in the binding site of DnmS. Images were made using PyMOL software.

In DnmS, the interactions with DNR include conventional hydrogen bonds W153, Q255/346, I257, T258/262 and E259 as well as hydrophobic interactions at T258, E259, F264 and W354/347. (Figure 8A). In addition, the interactions with TDP include conventional hydrogen bonds W153, I257, G326/328, T329, Q346, D349 and R352; hydrophobic interactions at T258; and a salt bridge at H324 (Figure 8B). The interactions analyzed above may be identified as potential candidate amino acids specific to DNR and TDP to understand substrate binding and glycosylation activities.

## 4. Discussion

Molecular chaperones, such as GroEL, GroES, DnaK, DnaJ, HptG and trigger factor (TF), are particularly useful for the expression of target proteins in *E. coli* [29]. Nearly 30% of *E. coli* proteins are reported to be aggregated without the assistance of chaperones [30]. Unlike enzymes with precise and finely tuned active sites, chaperones are heavy-duty molecular machines that work on a wide range of substrates and can assist nascent proteins to reach their native fold, protect subunits from heat shock during the assembly of complexes and prevent protein aggregation or mediate targeted unfolding and disassembly [30,31]. These molecular chaperones have a significant effect on the correct folding and assembly of target proteins in *E. coli* [19,32,33,34].

In this study, when the soluble expression of *dnmS* could not be obtained, we co-expressed it with a pGro7 plasmid containing the molecular partners GroEL and GroES, and a small amount of soluble expression could be obtained. The chaperone plasmid 7 was reconstructed by replacing the chaperone gene with one derived from *Streptomyces* and was co-expressed with DnmS, thereby, achieving an unprecedented high efficiency and soluble expression. This provides a strategy for the problem of the insoluble expression of GTs.

It is known that, for a large number of macrolide and aromatic biosynthesis GTs, the activity of GT requires accessory proteins. We already know that DnmS is involved in the biosynthesis of DNR in vivo. These are aromatic polyketide antibiotics with high cytotoxicity and are widely used in the chemotherapy of a variety of cancers. DnmS requires DnmQ to glycosylate the ε-RHO ketone to produce RHOD. In this study, the reverse GT activities of DnmS/DnmQ were first verified with an *E. coli* expression system in vitro.

We found that the enzyme activity of DnmS/DnmQ mixed protein was the highest, which was also verified by the study of homologous proteins of DnmS. For example, in the biosynthesis of the clinically important antibiotic erythromycin D, the GT EryCIII, in concert with its partner EryCII, attaches a nucleotide-activated sugar to the macrolide scaffold with high specificity [21]. In vitro studies of the GT/helper pair AknS/AknT in aclacinomycin biosynthesis showed both partners to be readily fusion-recombinant proteins when separately expressed, and AknT stimulated the glycosylation activity of AknS [35].

GT DesVII is involved in the biosynthesis of the macrolide antibiotics methymycin, neomethymycin, narbomycin and pikromycin in *Streptomyces venezuelae*. DesVII requires an additional protein component, DesVIII, for activity. The formation of the DesVII/DesVIII complex requires the co-expression of both genes in vivo and cannot be fully achieved by mixing the individual protein components in vitro. DesVIII assists the folding of DesVII during protein production and remains tightly bound during catalysis [36].

The above homologous protein enzyme activity analysis also shows the importance of GT and the helper protein complex for its catalytic activity. This paper preliminarily explored the reverse glycosylation reaction catalyzed by DnmS and found that the catalytic reaction has great versatility and practicability. This brings more possibilities for the further exploration of a variety of anthracycline antitumor drugs.

## 5. Conclusions

GTs are an important group of enzymes that catalyze the attachment of sugar moieties to acceptor molecules. Although there are over 800,000 putative GTs in the carbohydrate-active enzyme CAZy database, most of these proteins have never been purified, and their catalytic roles have not been verified. Thus far, more than 114 GTs presumably involved in the biosynthesis of microbial secondary metabolites have been identified [26,37,38]; however, few of them have been characterized in vitro. It is urgent to use *E. coli* expression systems for the soluble and efficient expression of GTs.

In summary, the catalytic characterization of DnmS and DnmQ in vitro was explored for the first time. Due to the construction of the novel molecular chaperone plasmid pGro7′ derived from *Streptomyces*, the glycosyltransferases DnmS demonstrated unprecedented high-efficiency and soluble expression in the *E. coli* expression system. This has guiding significance for the proteins in *Streptomyces* and will help to study the structure of these enzymes.

Then, the reverse glycosyltransfer reactions of DnmS and DnmQ and DnmS/DnmQ mixed protein were verified, and we found that DnmS/DnmQ mixed protein had the highest enzyme activity. Finally, the reverse glycosyltransfer catalytic characterization of DnmS indicated that the catalytic reaction of GT has great versatility and practicality. This presents new possibilities for the future exploration of diversified anthracycline antitumor drugs.

## Figures and Tables

**Table 1:** The bacterial strains and plasmids used in this work.

Strains or Plasmids	Relevant Characteristics	Reference or Source
*Escherichia coli*		
DH5α	F^−^ Φ80*lac*ZΔM15Δ(*lac*ZYA-*arg*F) U169 *rec*A1 *end*A1 *hsd*R17(r_k_^−^,m_k_^+^) *pho*A *sup*E44 *thi*-1 *gyr*A96 *rel*A1 λ^−^	Gibco BRL, Life Technologies
BL21 (DE3)	F^−^ *ompT hsdS*(r_B_^−^ m_B_^−^) *dcm*^+^ *gal*λ(DE3)	Novagen
BL21 Codon plus (DE3) RIL	F^−^ *ompT hsdS*(r_B_^−^ m_B_^−^) *dcm*^+^ *gal*λ(DE3) *end*A Hte [*argU ileY leuW* Cam^r^]	Novagen
22bS/DE3	BL21 (DE3) harboring 22bS, Amp^r^	This work
22bS/RIL	BL21 Codon plus (DE3) RIL harboring 22bS, Amp^r^, Cam^r^	This work
32aS/DE3	BL21 (DE3) harboring 32aS, Amp^r^	This work
32aS/RIL	BL21 Codon plus (DE3) RIL harboring 32aS, Amp^r^, Cam^r^	This work
32aS/pGro7	BL21 (DE3) harboring 32aS and pGro7, Amp^r^, Cam^r^	This work
32aS/pGro7′	BL21 (DE3) harboring 32aS and pGro7′, Amp^r^, Cam^r^	This work
22bQ/DE3	BL21 (DE3) harboring 22bQ, Amp^r^	This work
22bQ/RIL	BL21 Codon plus (DE3) RIL harboring 22bQ, Amp^r^, Cam^r^	This work
32aQ/DE3	BL21 (DE3) harboring 32aQ, Amp^r^	This work
32aQ/RIL	BL21 Codon plus (DE3) RIL harboring 32aQ, Amp^r^, Cam^r^	This work
Plasmids		
pET22b	Expression vector, C-terminal 6×His-tagged sequences, Amp^r^	Novagen
pET32a	Expression vector, N-terminal 6×His-tagged sequences, Amp^r^	Novagen
pGro7	Chaperone plasmid, Cam^r^	Takara
pGro7′	Chaperone plasmid with molecular chaperone gene of *Streptomyces*, Cam^r^	This work
22bS	pET22b containing *dnmS* coding region, Amp^r^	This work
32aS	pET32a containing *dnmS* coding region, Amp^r^	This work
22bQ	pET22b containing *dnmQ* coding region, Amp^r^	This work
32aQ	pET32a containing *dnmQ* coding region, Amp^r^	This work

**Table 2:** The primers used in this study.

Primer Name	Sequence
32a-dnmS-F	gccatggctgatatcggatccatgaaggtgctcgtgacggc
32a-dnmS-R	ctcgagtgcggccgcaagcttctagtgccggacgccctg
22b-dnmS-F	taagaaggagatatacatatgatgaaggtgctcgtgacggc
22b-dnmS-R	gtggtggtggtggtgctcgaggtgccggacgccctgccc
32a-dnmQ-F	Gccatggctgatatcggatccatgcccacacccacgtcc
32a-dnmQ-R	ctcgagtgcggccgcaagctttcacttctgggccagccg
22b-dnmQ-F	taagaaggagatatacatatgatgcccacacccacgtcc
22b-dnmQ-R	gtggtggtggtggtgctcgagcttctgggccagccgcag
groEL1-F	ttctcaaaggagagttatcaatggcgaagatcctgaagttcg
groEL1-R	ttggagctggtggtcgtcacgtgggagtggccgtggct
groES-F	gtgacgaccaccagctccaa
groES-R	atcttggccatcttctcgacgatcgcgagc
groEL2-F	gtcgagaagatggccaagatcatcgcgt
groEL2-R	ttctgcgaggtgcagggcaatcagaagtccatgtcaccaccc
pGro7-F	ttgccctgcacctcgcag
pGro7-R	tgataactctcctttgagaaagtccg

## Data Availability

Not applicable.

## Supplementary Materials

The following supporting information can be downloaded at: https://www.mdpi.com/article/10.3390/microorganisms11030762/s1, Figure S1: The expression of DnmS in different expression vector and host. Lane 1, 22bS/DE3 induced total protein; Lane 2, 22bS/DE3 induced supernatant; Lane 3, 22bS/RIL induced total protein; Lane 4, 22bS/RIL induced supernatant; Lane 5, 32aS/DE3 induced total protein; Lane 6, 32aS/DE3 induced supernatant; Lane 7, 32aS/RIL induced total protein; Lane 8, 32aS/RIL induced supernatant; M, protein marker (Genestar, 25, 35, 45, 65, 75, 100, 135 and 180 kDa.).
