# Supplementary material for: Expression and Purification of Glycosyltransferase DnmS from Streptomyces peucetius ATCC 27952 and Study on Catalytic Characterization of Its Reverse Glycosyltransferase Reaction
Source: Microorganisms. 2023 Mar 16;11(3):762. doi: 10.3390/microorganisms11030762 (PMC10058486; doi:10.3390/microorganisms11030762)
Supplement: Supplementary file 1 [file microorganisms-11-00762-s001.zip › microorganisms-2220756-supplementary.pdf]

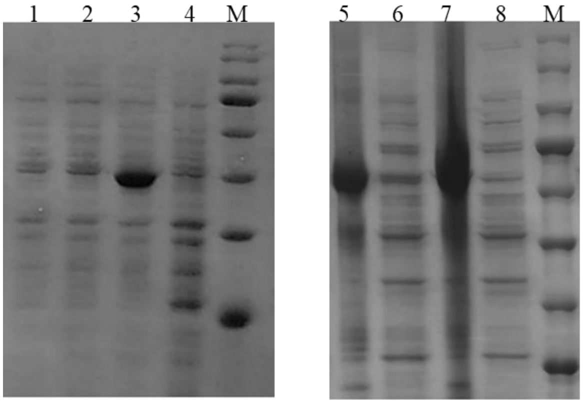


**Figure S1.** The expression of DnmS in different expression vector and host. Lane 1, 22bS/DE3 induced total protein; Lane 2, 22bS/DE3 induced supernatant; Lane 3, 22bS/RIL induced total protein; Lane 4, 22bS/RIL induced supernatant; Lane 5, 32aS/DE3 induced total protein; Lane 6, 32aS/DE3 induced supernatant; Lane 7, 32aS/RIL induced total protein; Lane 8, 32aS/RIL induced supernatant; M, protein marker (Genestar, 25, 35, 45, 65, 75, 100, 135 and 180 kDa.).
